# The Influence of Acitretin on Brain Lipidomics in Adolescent Mice—Implications for Pediatric and Adolescent Dermatological Therapy

**DOI:** 10.3390/ijms232415535

**Published:** 2022-12-08

**Authors:** Anna A. Lauer, Vu Thu Thuy Nguyen, Daniel Janitschke, Malena dos Santos Guilherme, Cornel M. Bachmann, Heike S. Grimm, Tobias Hartmann, Kristina Endres, Marcus O. W. Grimm

**Affiliations:** 1 Deutsches Institut für Demenzprävention (DIDP), Neurodegeneration and Neurobiology, Saarland University, 66421 Homburg, Germany; 2 Experimental Neurology, Saarland University, 66424 Homburg, Germany; 3 Nutrition Therapy and Counseling, Campus Rheinland, SRH University of Applied Health Sciences, 51377 Leverkusen, Germany; 4 Department of Psychiatry and Psychotherapy, University Medical Center Johannes Gutenberg-University, 55131 Mainz, Germany

**Keywords:** acitretin, adolescence, brain development, retinoid, lipidomics, triglycerides, phosphatidylcholine, plasmalogens, carnitines, lyso-phosphatidylcholine

## Abstract

Administration of systemic retinoids such as acitretin has not been approved yet for pediatric patients. An adverse event of retinoid-therapy that occurs with lower prevalence in children than in adults is hyperlipidemia. This might be based on the lack of comorbidities in young patients, but must not be neglected. Especially for the development of the human brain up to young adulthood, dysbalance of lipids might be deleterious. Here, we provide for the first time an in-depth analysis of the influence of subchronic acitretin-administration on lipid composition of brain parenchyma of young wild type mice. For comparison and to evaluate the systemic effect of the treatment, liver lipids were analogously investigated. As expected, triglycerides increased in liver as well as in brain and a non-significant increase in cholesterol was observed. However, specifically brain showed an increase in lyso-phosphatidylcholine and carnitine as well as in sphingomyelin. Group analysis of lipid classes revealed no statistical effects, while single species were tissue-dependently changed: effects in brain were in general more subtly as compared to those in liver regarding the mere number of changed lipid species. Thus, while the overall impact of acitretin seems comparably small regarding brain, the change in individual species and their role in brain development and maturation has to be considered.

## 1. Introduction

Acitretin, a second-generation retinoid, is indicated for the systemic treatment of severe psoriasis in adults, achieving approval from the FDA already in 1997 [1]. The mode of action of retinoids, including acitretin, is still not entirely understood. Modulation of epidermal differentiation, proliferation, and immunomodulatory effects have been described. Unlike most systemic psoriasis treatments, acitretin has not been found to act immunosuppressive and therefore can be used in already immune-deprived or vulnerable patients (e.g., [2]). However, pediatric use of acitretin has not been approved yet due to lack of clinical trials. The prevalence of psoriasis in children ranges from 0 (Taiwan) to 2.1% (Italy) but this might be an underestimation due to the occurring mild/atypical forms [3]. It increases from 1 year to the age of 18 [4]. Especially in children and young adults, the psychological burden of low self-esteem related to the visible skin lesions has to be taken into account [5]. This might even explain the increased risk of psoriatic children for mental illness-like depression and anxiety in comparison to healthy controls [6].

Prescription of acitretin is strictly forbidden during pregnancy. Only two cases of fetal exposure have been published in fact and one report with postnatal follow-up [7,8,9]. However, investigation of the embryopathy caused by retinoids in general has unraveled a wide range of severe effects in the fetus including not only cleft-palate or thymus defects but also optic nerve and CNS abnormalities (reviewed in [10]). In retrospective studies or case studies on children, mostly the typical adverse events (AE) that are similarly reported for adults have been observed. A retrospective case note review on a UK population including 174 patients at the mean age of 8 seen between 1993 and 2015 identified clinical AEs in 24% and laboratory AEs in 22% of participants [11]. This led to permanent cessation of 10 and 4%. A study from North America/Europe including 390 children aged approximately 11.4 years at the start of treatment found 67% to develop one or more AEs in the acitretin-receiving group (14.6% of the study population, [12]). Mostly, the AEs belonged to the categories skin/hair such as skin fragility or hair loss; however about 10–14% of the patients were showing hyperlipidemia [11,12]. The frequency is low as compared with adult patients. A study with psoriatic patients aged 45 to 48 years, revealed about 40% of patients having elevated laboratory values for triglycerides more than 20% higher than the reference range maximum [13]. The discrepancy between pediatric and adult patients might be due to the plausible lack of relevant comorbidities for metabolic effects in the young group such as alcohol-abuse or diabetes mellitus or a different lifestyle including higher physical activity in general. This might erroneously result in the assumption that alterations in lipid profile do not need to be of major concern in pediatric patients receiving acitretin. Nevertheless, considering the immense importance for lipids in establishing and preserving brain function, this has to be a special focus of therapeutic monitoring in young patients where development of the brain is still ongoing. From the embryonic to postnatal states, even small deviations from optimal supplementation with certain lipid species may influence growth and maturation of the brain as shown in the following for polyunsaturated fatty acids (PUFAs). For example, it has been shown that placental supply of docosahexaenoic acid (DHA) is obligatory in particular in human third trimester for brain development of the growing fetus [14]. Perinatal DHA supplementation reduced the risk of lower IQ scores in children from very low-income families [15,16]. Administering high DHA content to children at the age of 9 months led to higher attention [17] and supply of children aged 3 to 13 years with DHA and eicosapentaenoic acid (EPA) resulted in a significant increment of academic performance [18]. Similar observations regarding the important role of lipids for brain development have been made in rodent models: e.g., postnatal dietary deprivation of parent mice elicited schizophrenia-like phenotypes in the offspring at adulthood [19]. Moreover, mouse pups raised by transgenic dams with elevated alpha lipoic acid (ALA) and EPA levels in milk achieved a 1.6-fold-elevated DHA amount in brain and higher visual placement scores [20].

**Figure 1:**
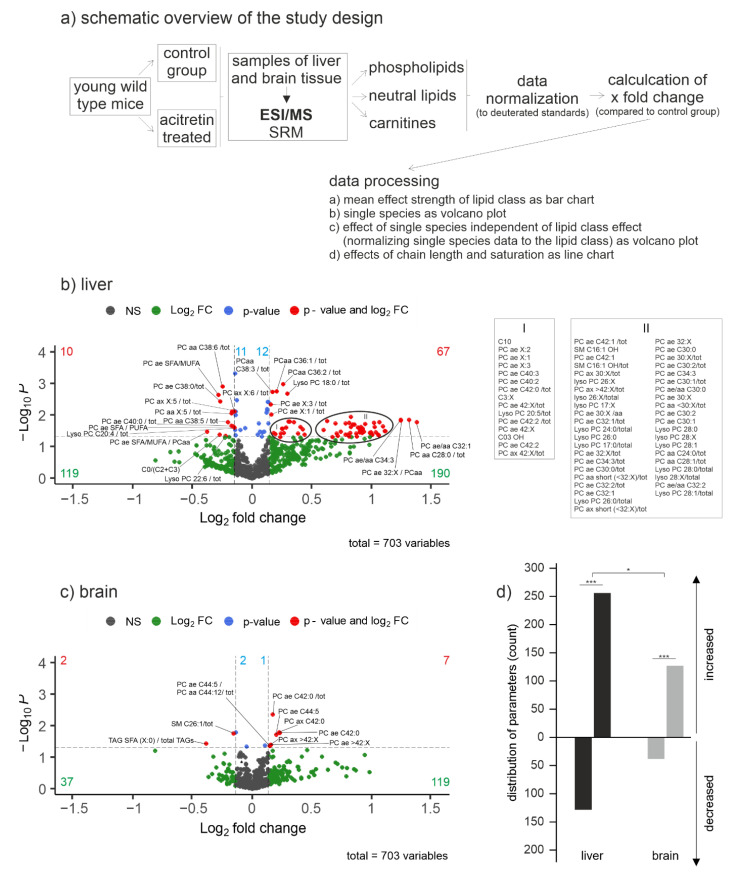
Lipid changes in acitretin-treated wild type mice compared to controls. Mice were treated starting from the age of 9 weeks with seven daily acitretin i.p. injections. One liver lobe and one hemisphere of the brain were harvested for lipid extraction and analysis. A schematic overview on the study design and data processing is shown (**a**). Fold changes of the included parameters are plotted logarithmically against the *p*-value (−Log10) for liver (**b**) and brain tissue (**c**) of female C57Bl6/J OlaHsd mice (n = 6 per group). Lipid species without significant changes are represented as grey dots, species with a fold change greater than the average SEM as green dots. Species with a significant fold change (*p* < 0.05) are indicated in blue and those with a *p*-value < 0.05 and a fold change greater than the SEM as red dots in the volcano plot. The exact number of changed parameters in every part of the volcano plot is provided in the appropriate color. For reasons of clarity and comprehensibility, names of lipid species from high density regions of lipids in liver have been indicated in the text besides the graph (I, II). Parameters that were significantly changed after adjustment of *p*-value to multiple testing are provided in Supplementary Figure S1. Distribution of parameters with a fold change greater than the average SEM represented as number of de- and increased parameters in brain and liver tissue can be found in (**d**). Statistical significance of the observed numbers in de- and increased parameters in brain or liver tissue was calculated using binomial test to check whether the frequency distribution of lipids was changed between control and treatment and between tissues (*, *p* < 0.05; ***, *p* < 0.001).

Due to the lack of data especially on early life impact of acitretin on brain lipid profile, we set up a study to investigate lipidomics of brain tissue from mice aged 9 weeks at the start of the treatment. This age was chosen as it represents a late phase of brain development in mice. Sexual maturity of mice is achieved with 8–12 weeks; however, brain development is still ongoing: myelination still increases up to the age of three months and cortex continues to flatten [21]. Full brain weight of the rat is reached with about 60 days, while mature PFC is seen only with 90 days postnatal [22]. This together with a calculation following the suggestion from Dutta and Sengupta [23] leads to the assumption that 9–10 week-old mice maybe mirroring an age of late adolescence to early adulthood. Mice were treated with 10 mg/kg, equalizing a human equivalence dosage of 0.81 mg/kg (based on [24]) and thus referring to the start dosage used in pediatric patients (0.3–1.0 mg/kg, [10,25]). Brains were extracted after sub-chronical treatment and subjected to shotgun mass spectrometry lipidomics. Liver tissue served as a peripheral control and a proxy to potential human hepatic hyperlipidemia. Shotgun mass spectrometry lipidomics, which is described in more detail in Section 4.3.4, was used as it allows the simultaneous semi-quantitative measurement of a variety of lipid species. The analyzed lipids in this study included species from different lipid classes: phospholipids (43 phosphatidylcholine species, 39 phosphatidylcholine plasmalogen species, 22 lyso-phosphatidylcholine species, 15 sphingomyeline species), neutral lipids (17 triacylglyceride species) as well as carnitine, acyl-carnitine, and acetyl-carnitine (41 species in total). As shown in the schematic on the design of the study (Figure 1a), the obtained data were normalized by utilizing deuterated standards and the fold change of the acitretin-treated group compared to control group was calculated. Furthermore, the individual lipid species were normalized to the levels of the corresponding lipid class to highlight potential changes in the distribution of the lipid species. This type of normalization allows fatty acid distributions in the lipid class to be detected independently of potential changes in the lipid class per se.

## 2. Results

### 2.1. Global Effect of Acitretin on Liver and Brain Lipids in Young Wild Type Mice

The available case studies on human children and adolescent patients already suggested that lipid metabolism is affected by acitretin administration. This could be confirmed for analysis of mice with onset of treatment within the 9th week of age (Figure 1b). From all analyzed parameters, 10 were decreased in liver with a *p*-value < 0.05 and a fold change greater than the average SEM. Even more lipids were increased (67) according to these quality criteria. As small changes in distinct lipid species might already affect bio-membrane architecture or cell signaling, it has to be recognized that in total, about 100 lipid species were decreased and 200 increased with a *p* > 0.05 and an effect strength > SEM. For brain, only two lipid levels decreased, achieving a *p* < 0.05 and the respective fold change size and seven increased (Figure 1c). Nevertheless, 37 lipids were found decreased and 119 increased with a *p* > 0.05 and an effect strength > SEM. In sum, not only liver is affected in means of lipid metabolism, but both organs showed a distinct acitretin-induced change in patterns of lipid species (Figure 1d).

### 2.2. Impact of Acitretin on Liver Lipid Metabolism in Young Wild Type Mice

The liver is the organ that mainly orchestrates lipid metabolism and serves as a source of various lipids and lipid building blocks for other organs. Moreover, hyperlipidemia as assessed by blood samples is mostly based on derangement of liver function. Therefore, and to gain comparison to a potentially altered brain lipid homeostasis, we first investigated liver tissue of acitretin-treated young mice in higher detail.

#### 2.2.1. Effect of Acitretin on Selected Lipid Metabolism Genes in the Liver

**Figure 2:**
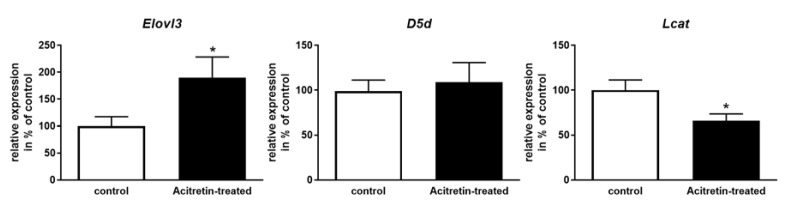
Quantitation of the mRNA level of selected lipid metabolism genes. Mice were treated as described (Figure 1) and one liver lobe harvested for RNA preparation. RNA was subjected to qRT-PCR with Elovl3-, D5d- or Lcat-specific primers. Transcript levels of Gapdh were used for normalization. Data are presented as mean + SEM (n = 6 for Elovl3 in acitretin-treated mice, n = 7 for all other groups; Student’s unpaired *t*-test; * *p* < 0.05).

More than 4000 of retinoic acid receptor α (RARα) binding genes have been shown to be bound by RXRα in murine liver and occupied the majority of total RXRα bindings, followed by PPARα, FXR, LXR, and PXR [26]. This indicates that retinoic acid-related signaling must have an important function in liver metabolism regulation. Acitretin is known to deliberate retinoic acid from its cellular binding protein CRABP (cellular retinoic acid binding protein) via higher binding affinity [27]. Thus, the synthetic compound elevates the abundancy of the bio-reactive ligand. Heterodimers of RXRα with the respective nuclear receptors have a great impact on lipid processing pathways, for example, RXRα-PPARα distinctly binds to the genes of fatty acid metabolic processes. In this example, RXR serves as a permissive partner, meaning that it responds to its ligand retinoic acid within the dimer [28]. Within a first step, we therefore analyzed expression of three representative lipid metabolism genes in the liver of acitretin-treated young mice that have the theoretical potential to react to the treatment (Figure 2). Elovl3, a gene encoding an enzyme (elongation of very long chain fatty acids protein 3) that elongates saturated and monounsaturated fatty acids, contains three peroxisome proliferator activated receptor (PPAR)-responsive elements within the promoter region [29]. Delta5 fatty acid desaturase (D5d; Fat-4) mRNA was increased by fenofibrate, an agonist of PPARα [30]. Finally, β-carotene-15,15’-oxygenase-deficient mouse embryos, that are incapable of cleaving β-carotene to obtain retinoids, displayed a reduced mRNA level of lecithin-cholesterol acyltransferase (Lcat; [31]).

While Elovl3 transcription was increased to 190% of control animals and Lcat mRNA level was decreased to about 65% upon acitretin administration, expression of D5d was unaltered (*p* = 0.69; Figure 2). This indicated that various lipid metabolism functions such as chain elongation and esterification of cholesterol might be affected, but the impact of acitretin is not globally seen for all putative retinoid targets.

#### 2.2.2. In-Depth Mass Spectrometry Analysis of Lipid Species in Liver Tissue of Acitretin-treated Young Mice

For identification of lipid classes and single lipid species that are affected by acitretin treatment, we analyzed triacylglycerides (TAG), phosphatidylcholine (PCaa), plasmalogens (PCae), lyso-phosphatidylcholine (lyso-PC), sphingomyelins (SM), and carnitines. Observed alterations of a single lipid can be based on the head group or due to the fatty acid (FA) bound. Therefore, each lipid species was normalized to the average lipid class effect to indicate the contribution of the respective FA as described before [32]. By normalization to total lipid class, the alteration in the distribution of single lipid species in relation to their respective lipid class can be evaluated and it can be deduced if the single lipid changes independently of an alteration of the lipid class in general. All data described below were gained by *t*-test analysis. For results after false discovery rate (FDR) correction, see Supplementary Table S1.

- TAG

**Figure 3:**
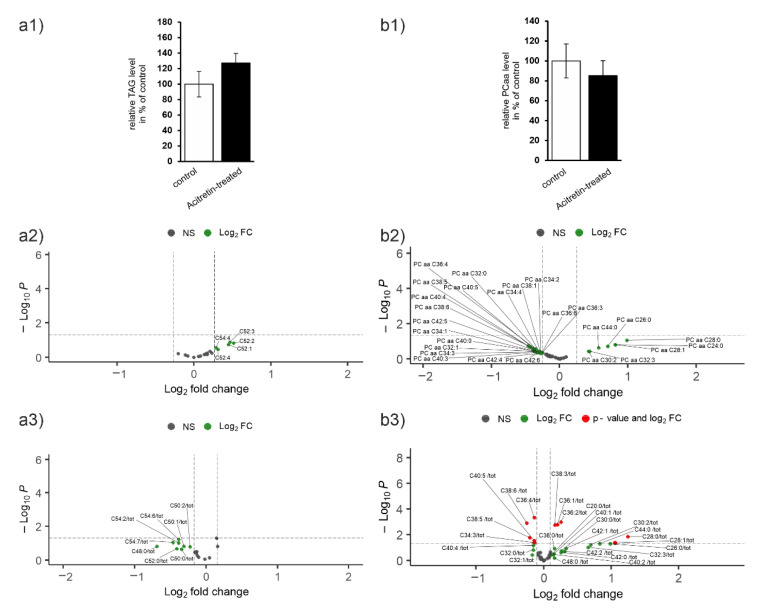

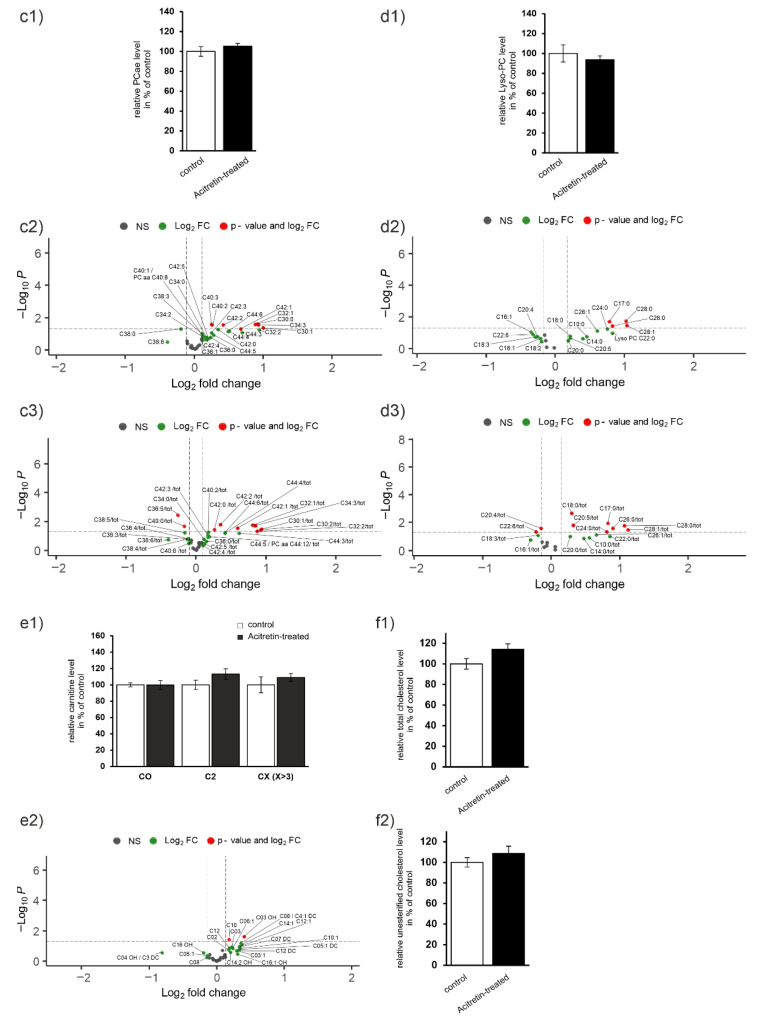
Lipid species in hepatic tissue. Mice were treated as described (Figure 1) and lipids from liver lobes extracted by combination of mechanical and chemical treatments. Lipid species were identified by mass spectrometry: (**a**) triacylglycerides (TAG), (**b**) phosphatidylcholine (PC aa), (**c**) plasmalogens (PC ae), (**d**) lyso-PC, (**e**) carnitines (1: relative change of groups; 2: individual carnitines), and (**f**) total cholesterol (**f1**) and unesterified cholesterol (**f2**). Sphingomyelins (SM) can be found in Supplementary Figure S2 and effect sizes for all significantly affected species in Supplementary Figure S3. Subfigures (**a1**–**d1**) display change in % of the whole lipid class. Figures (**a2**–**d2**) show individual lipids and (**a3–d3**) individual lipids normalized to the whole lipid class. Statistical significance of the mean in liver tissue of acitretin-treated compared to control mice was calculated using two-sample *t*-test (n = 6 animals per group).

Figure 3a illustrates the analysis of TAG species. In sum, the amount of hepatic TAGs increased to 127% of control-treated animals (Figure 3a1). We furthermore observed that from 17 TAG species none, in general, decreased and five increased (Figure 3a2). Normalization of the single species to total TAG amount revealed that many of the 17 lipid species showed an effect regarding lowered relative abundance with TAG 48:0 displaying the strongest decrease (reduced to about 62%, Figure 3a3). Nevertheless, none of the changes reached statistical significance.

- PC aa

Expansion of lipid droplets to accommodate newly synthesized neutral lipids requires an increase in phospholipid content. Diacyl-phosphatidylcholines (PC aa) are the major phospholipid species in the monolayer surrounding lipid droplets, followed by phosphatidylethanolamine-containing lipids. However, here, we found a small decrease in the total amount of PC aa due to acitretin administration (Figure 3b1). Nineteen species decreased with an effect size > SEM and seven increased when data were not normalized (Figure 3b2). After normalization to the corresponding lipid class, five decreased lipid species showed a significant effect (PC aa C36:4/38:6/40:5/38:5/34:3) and five elevated species (C38:3/C36:1/36:2/28:0/26:0; Figure 3b3).

- PC ae

While the distinct function of plasmalogens still needs to be unraveled, it seems that they play important roles in protection against oxidative stress due to their substitution (ether vinyl instead of FA; [33]) and in bio-membrane architecture (reviewed in [34]). Moreover, correlation to multiple disorders such as Alzheimer’s disease and comparably high expression in human brain suggest an important neuronal function even if synthesis is located to liver [35]. The majority of the 39 investigated PC ae revealed an increase, while only two molecular entities could be identified to be decreased after normalization to suffice *p*-value and effect size criterion (Figure 3c2,c3: PC ae C40:0 and PC ae C36:5). Seven species were elevated even if total amount of PC ae was only marginally increased to 105% of control (Figure 3c1).

- Lyso-PC and SM

Lyso-PC has been assigned a potential multitude of cellular signaling functions and it has been proposed to act as an agonist for the platelet-activating factor (PAF) receptor, the G2A sphingophosphorylcholine receptor, and the GPR4 sphingophosphorylcholine receptor [36]. It also presents a “find me” signal in apoptosis and recruits phagocytes to sites of damage [37,38,39]. Within the brain it has been noted to impair the barrier function of the endothelium in the microvasculature, to induce inflammation, and to elicit oligodendrocyte demyelination. Synthesis of the signaling molecule mostly occurs in the affected tissue but it also is one of the major transport forms for lipids.

Analysis of liver content of lyso-PC indicated a small decrease in acitretin-treated mice (about 6%, Figure 3d1). From 22 analytes, six were decreased upon acitretin administration and 11 increased (Figure 3d2). After normalization, lyso-PC C22:6 and C20:4 were the only lyso-PCs that still could be identified as decreased (*p* < 0.05 and fold change > SEM). Seven lyso-PCs occurred increased when related to total lyso-PC content, e.g., lyso-PC C28:1 (Figure 3d3).

From the 15 analyzed SM species, only two provided a significant *p*-value after the normalization (SM C24:0 decreased and C16:1 increased, Supplementary Figure S2). Additionally, the effect on total SM content was comparably small (6% decrease) so that the impact of acitretin on this lipid class in the liver of mice at the respective age seems rather restricted.

- Carnitins

Acylcarnitines are the transport form of FAs when they are introduced into the mitochondria. While free carnitine amount was not altered due to acitretin-treatment (Figure 3e1), acetylcarnitine and molecules carrying propionyl moieties or longer acyl chains (CX, X > 3) were elevated (13 and 9%) when evaluated in sum. This might point at changes in β-oxidation. On the species level, four carnitines were found decreased while the majority increased (16 of 41; Figure 3e2). Two of those correlated to a *p*-value < 0.05 and an effect size higher than the average SEM (C10 and C03 OH).

- Cholesterol

Both, total cholesterol and free, unesterified cholesterol were increased by acitretin-administration in young mice (Figure 2 and Figure 3f1; 14 and 8%). Cholesterol is not only an important building block for steroid synthesis, it also regulates membrane fluidity and by this or by direct binding affects properties of membrane-embedded proteins. Especially, the increase in unesterified cholesterol might be due to the observed decrease in liver Lcat mRNA amount (Figure 2) as this enzyme catalyzes esterification of cholesterol and fatty acid of lecithin. However, statistical significance was not reached (*p* = 0.174 and 0.360).

- Saturation and chain length

The function of a respective lipid is not only determined by its head group but also dominantly by saturation of the attached FAs and their chain length (for an example see: effect of chain length and saturation on sodium-glucose cotransporter; [40]).

**Figure 4:**
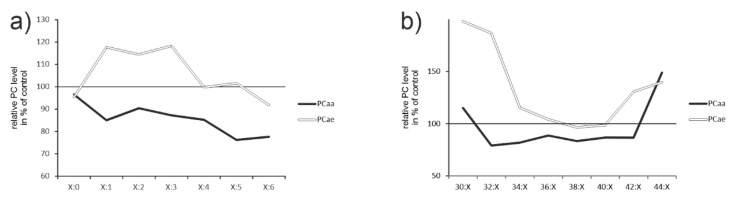
Saturation and chain length of phosphatidylcholines in liver tissue from acitretin-treated mice. Processing of the data regarding saturation (**a**) and chain length (**b**) included the following species: PCaa saturation: C20-48:0, C28-42:1, C30-42:2, C32-40:3, C34-42:4, C36-42:5, C36-42:6; PCaa chain length: C30:0-2; C32:0-3; C34:1-4; C36:0-6; C38:0-6; C40:0-6, C42:0-6; C44:0; PCae saturation: C30-42:0; C30-42:1, C30-42:2; C34-44:3; C36-44:4; C36-44:5; C38-44:6; PCae chain length: C30:0-2; C32:1-2; C34:0-3; C36:0-5; C38:0-6; C40:0-6; C42:0-5; C44:3-6. These data could indicate potential effects of acitretin on desaturases or peroxisomes.

In general, desaturation decreased when comparing tissue from control-treated animals with acitretin-treated ones (Figure 4a). However, the amount of PCae with up to three double bonds increased. The amount of shorter (30:X) and acyl residues longer than 42 increased on the expense of species within intermediate chain length (Figure 4b). Here, the effect was most pronounced for PCae. However, PCae only make up a small amount compared to the other lipids within biological membranes and thus, increase in PCaa species with chain length >42 might be more relevant for the resulting physical state of the cellular membrane. Together with the observed increase in cholesterol (Figure 2 and Figure 3f1), an increased stiffness has to be assumed and chain length effects of PCaa might be explained by altered expression of respective elongases (e.g., Elovl3, Figure 2).

### 2.3. Impact of Acitretin on Lipid Metabolism of the Brain

Lipids are essential components for the structure but also for the function of the brain. Only adipose tissue has higher lipid content than the brain, where lipids constitute a high proportion of the organ weight (e.g., 73 mg/g in rat hippocampus; [41]). While adipose tissue largely stores lipids in the form of triglycerides for energy conservation or delivery to other tissues, the brain is thought to mainly utilize acylic lipids to generate phospholipids for cellular membranes. Some FAs can be synthesized de novo within the brain, but the majority has to be transported into the brain from the systemic circulation. The observation of liver-related neurons in key hypothalamic nuclei allows speculation that these neurons link central lipid sensing and hepatic lipid metabolism (reviewed in [42]). Therefore, a bidirectional influence between brain and liver has to be assumed in lipid metabolism.

- TAG

**Figure 5:**
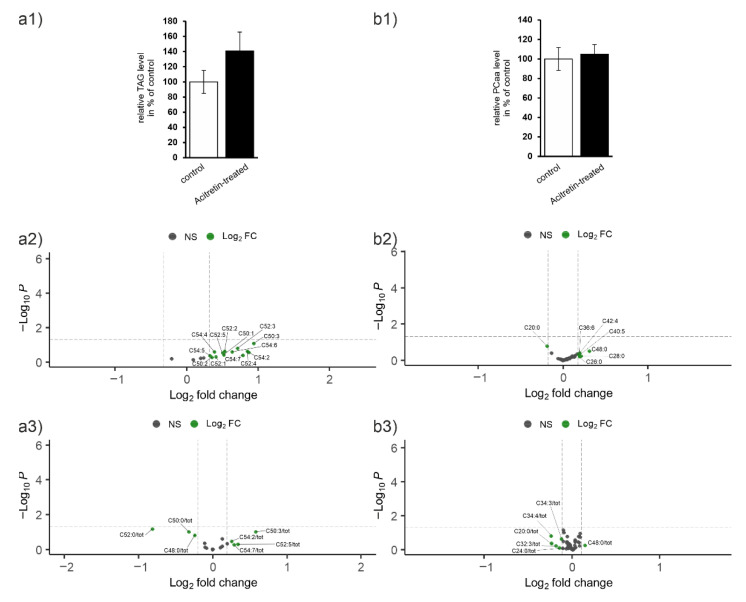

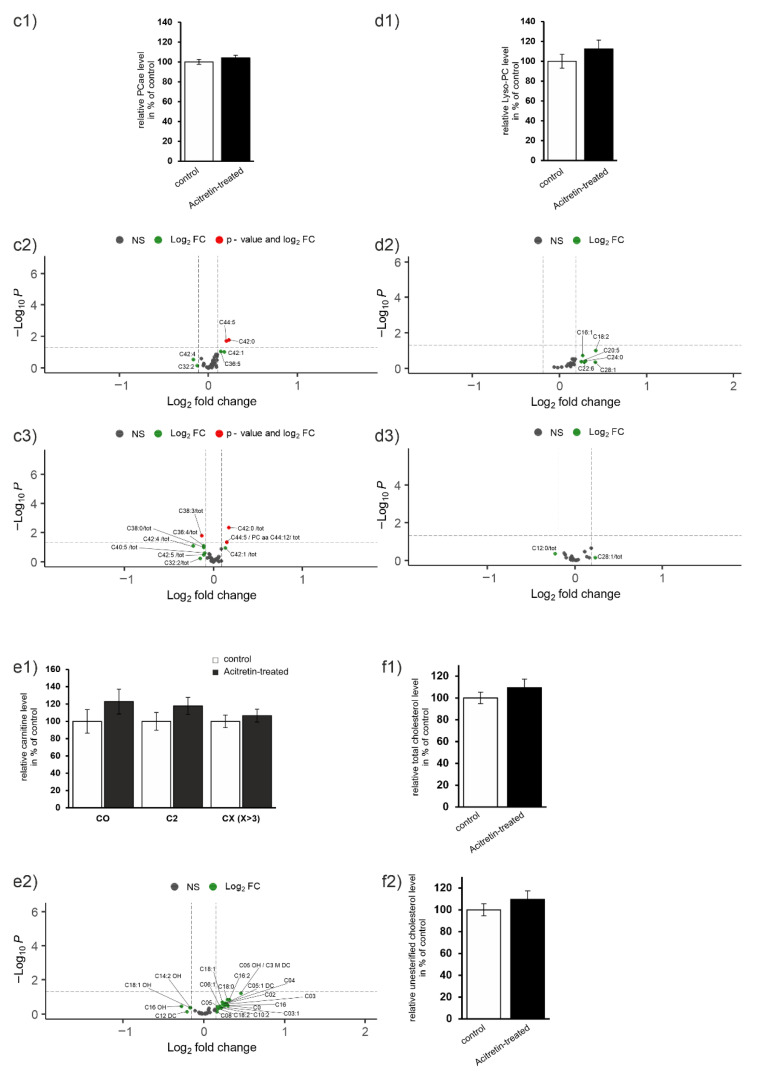
Lipid species in brain parenchyma of wild type mice treated with acitretin. Mice were treated as described (Figure 1) and lipids from total brain extracted by combination of mechanical and chemical treatments. Lipid species were identified by mass spectrometry: (**a**) triacylglycerides (TAG), (**b**) phosphatidylcholine (PC aa), (**c**) plasmalogens (PC ae), (**d**) lyso-PC, (**e**) carnitines (1: relative change of groups; 2: individual carnitines), and (**f**) total cholesterol (**f1**) and unesterified cholesterol (**f2**). Sphingomyelins (SM) can be found in Supplementary Figure S4 and effect sizes for all significantly affected species in Supplementary Figure S5. Subfigures (**a1**–**d1**) display change in % of the whole lipid class. Figures (**a2**–**d2**) show individual lipids and (**a3**–**d3**) individual lipids normalized to the whole lipid class. Statistical significance of the mean effects in liver tissue of acitretin-treated mice compared to control mice was calculated using two sample *t*-test (n = 6 animals per group).

When analyzing TAG from brain parenchyma, similar results were obtained as for liver: the overall amount increased to 141% (Figure 5a1). In general, when considering non-normalized data, an increase in most species was observed but did not survive normalization to lipid class with a respective *p*-value (Figure 3 and Figure 5a2).

- PCaa

PCaa level was unaltered between control- and acitretin-treated mice (Figure 5b1). Only one species was found decreased and five increased (Figure 5b2). In contrast to liver, none of these showed a *p* < 0.05 after normalization (Figure 5b3), emphasizing that there was not an additional FA-dependent effect and alteration was more a distributional effect within the lipid group.

- PC ae

Total amount of PC ae was marginally increased (Figure 5c1) to 104%. Individually, only one lipid species could be found to be statistically significantly decreased (C38:3; Figure 5c2) while three were found to be increased after normalization (C42:0/C44:5 and C44:12; Figure 5c3).

- Lyso-PC and SM

Analysis of liver content of lyso-PC species indicated an increase in acitretin-treated mice (about 12%, Figure 5d1), which deviates from the decreased amounts found in liver. From 22 analytes, six were increased upon acitretin administration and none found to be decreased (Figure 5d2). After normalization to lipid class, none of the changes observed for single species remained (Figure 5d3).

From the 15 analyzed SM species, only one was found to be changed after the normalization and filtering for *p* < 0.05 (SM C26:1 decreased; Supplementary Figure S4). Additionally, the effect on total SM content was higher and in the other direction of effect as observed for liver (11% increase) so that the effect of acitretin on this lipid class in the murine brain at the respective age seems rather more pronounced than that within liver.

- Carnitins

Free carnitine amount was increased due to acitretin-treatment (Figure 5e1; 23%), and acetylcarnitine and molecules carrying propionyl moieties or longer acyl chains (CX, X > 3) were elevated (18 and 7%). On the species level, the multitude of carnitines was found increased (16 of 41; Figure 5e2), while only four decreased. However, none of the single species correlated to a *p*-value < 0.05 and an effect size higher than the average SEM.

- Cholesterol

Both, total cholesterol and free, unesterified cholesterol were increased by acitretin in brain tissue derived from young mice (Figure 2 and Figure 5f1; about 9% each) but without reaching statistical significance.

- Saturation and chain length

In contrast to liver, chain length and saturation degree were only very subtly affected in brain with no clear directionality. The same holds true for saturation degree (Supplementary Figure S6).

Finally, an enrichment analysis was performed using the lsea function from the R package “lipidr” [43] (R package version 2.10.0, https://github.com/ahmohamed/lipidr; accessed on 20 November 2022) on data sets for both tissues (see Supplementary Figure S7a). The enrichment analysis revealed that in liver, PCaa was statistically significantly decreased after acitretin-treatment, which is in line with Figure 3b1, in which this lipid subgroup also was found decreased. However, enrichment analysis, which examined chain length and saturation only showed non-significant alterations (Supplementary Figure S7b,c). Effect strength of chain length and saturation were further visualized in a heat map (Supplementary Figure S7d,e).

## 3. Discussion

Lipids play an important role within the brain, not only as building blocks for cellular membranes but also as signaling molecules. The metabolism of this substance class istightly regulated for maintenance of neuronal structure and function but also to transfer information about metabolic processes between the central nervous system and peripheral organs such as the liver (reviewed in [42]). The brain is not only enriched in lipids but also contains highly specific lipid species, which even can be used for characterization of certain cell types of the brain or brain areas. For example, cultured murine neurons comprised elevated levels of PC, PE, cholesterol, and ceramide, while astrocytes displayed high levels of phosphatidylserine, phosphatidylinositol, and diacylglycerol and microglia showed, e.g., high levels of sphingomyelin [44]. Puberty of rodents and humans is characterized by an intense reformation of certain brain areas and networks (e.g., [45,46]). Therefore, puberty and early adulthood present an especially vulnerable period for changes in lipid metabolism, as for instance shown by the effect of high fat diet-induced precocious puberty on neurodevelopment in mice [47]. Consequently, drugs interfering with lipid homeostasis have to be cautiously considered when administered to infants and adolescents. Acitretin applied with the same treatment regimen as we used here, revealed in Alzheimer’s disease model mice aged 30 weeks that from 750 investigated lipid parameters, 114 tended to increase whereas 176 parameters tended to decrease in brain [32]. In total, number of lipid parameters affected by the drug within brain was comparable to that observed in liver of the adult disease model mice. However, direction of effect and affected lipid species were tissue-specific: for example, TAG increased in liver by about 15% while they were found reduced to 90% in brain as compared to control-treated mice. In adolescent wild type mice, we observed a slightly different picture: number of affected lipids was decreased in brain and effects seemingly were even milder in brain (nine lipids significantly affected with sufficient effect strength in brain, 77 lipids affected in liver). Aging and certain disorders such as Alzheimer’s disease lead to a reduced bioavailability of retinoic acid [48,49,50]. Acitretin acts not directly as a ligand for retinoic acid but allows release of the bioactive retinoic acid from its intracellular binding proteins [27]. Thus, it seems contra-intuitive that within a younger organism—probably having higher intracellular retinoic acid levels stored—the effect is smaller than in an aged and disease-impaired animal. The cytochrome P450 (CYP)26 family enzymes CYP26A1, CYP26B1, and CYP26C1 exert retinoid metabolism in humans (reviewed in [51]). A 3.5% decline in CYP enzyme content for each decade of life has been reported in humans already starting from the age of 40 onwards but also renal blood flow, renal filtration, or body composition is optimal in young individuals and then decreases with age (e.g., [52]). Similar observations have been made in mice [53] and thus might hint at a faster degradation of retinoic acid and probably acitretin itself in younger individuals, finally resulting in limited effects.

Group analysis of lipid species in tissue after acitretin-administration revealed that some were simultaneously affected in liver and brain of young mice such as cholesterol (increase) or PC ae (increase), while others were oppositely regulated (lyso PCs: decrease in liver, increase in brain). In sum, all observed group changes were non-significant. This might—in addition to the low number of individual lipids that were affected—lead to the assumption that acitretin-treatment unhesitatingly can be applied to infants and adolescents. However, our study is limited due to the usage of whole brain tissue specimen. It cannot be excluded that the effect is much more pronounced when local distribution would be taken into account. Moreover, lipid metabolism is mostly tightly regulated and thus, also small changes might have a deleterious outcome. Retinoic acid has been described to inhibit glucosylceramide synthase and ceramidase, which both would lead to blockade of ceramide degradation (summarized in [54]); however, retinoic acid also activates sphingomyelinase, leading to conversion of sphingomyelin to ceramide. This would, theoretically lead to decreased sphingomyelin levels and increase in ceramides. Nevertheless, sphingomyelins were increased in the brain of young mice (111%), while in old Alzheimer’s disease model mice, we indeed observed the theoretically expected reduction [32]. This suggests that in younger mice other enzymes in the sphingolipid pathway, like the serine palmitoyl transferase (SPT), might be elevated by acitretin. Ceramides that can be formed from sphingomyelins have a tendency for self-association and segregate into specialized micro-domains, promoted by the small polar head group of these lipids [55,56]. Already small amounts of ceramides suffice to induce these changes in membrane topology [57]. Interestingly, sphingomyelin treatment resulted in increased proliferation, maturation, and differentiation of oligodendrocyte precursor cells and increased axon myelination in Sprague Dawley rat pups [58]. Additionally, higher levels of sphingomyelin in infant nutrition products were associated with pronounced verbal development in very young infants [58] and associated with early myelination trajectories [59]. Therefore, the slight increase in SM might be even beneficial for a brain during a re-organization phase.

Comparably, obvious increases were also found for carnitines and lyso PCs in the brain of young mice, while not apparent in liver. Carnitine and its acetylated derivatives elicit improved energy status and ameliorate the detriments obtained in pediatric brain damage models such as oxidative stress [60]. These molecules may provide acetyl-CoA for energy generation or for acetylcholine synthesis, be incorporated into neurotransmitters such as glutamate or GABA, and into lipids needed for myelination and cell growth (summarized in [60]). Lyso PC has been reported to act neuroprotective in transient global ischemia in adult rats and in glutamate-evoked excitotoxicity in primary cultures of cerebellar granule cells [61]. While it has been described that lyso-PC is, for example, decreased in Alzheimer’s disease patients brains and in tissue of mouse models [62,63], we were not able to identify reports on its distinctive role in late brain development. Higher serum levels of acyl-lyso-PC and alkyl-lyso-PC were associated with higher weight, length, and head circumference in infants at birth [64], probably pointing at a beneficial effect. The cytosolic calcium-dependent phospholipase A2 (cPLA2) seems to preferably target phosphatidylcholine, resulting in release of lyso-PC and arachidonic acid (ARA) (for a review on PLA2: [65]). We have not assessed the amount of free ARA; however, it can be assumed that an increase in lyso-PC will be accompanied by it. ARA can be metabolized by cyclooxygenases, lipoxygenases, and CYPs, resulting in oxygenated products such as prostaglandins. These products are known to mediate inflammatory processes and thus might attribute negative interpretation of increased lyso-PC amounts after acitretin administration.

Only 12 single lipids passed filtering for significant effect size in the brain when comparing acitretin-treated and control-treated animals: PC ae C42:0; PC ae C44:5; PC ae C38:3/tot; PC ae C42:0/tot; PC ae C44:5/PC aa C44:12/tot; PC ae >42:X; PC ae 38:X/tot; PC ae >42:X/tot; PC ax >42:X; PC ax C42:0; SM C26:1/tot; TAG SFA (X:0)/total TAGs (all increased despite the two last ones; for a summary see Supplementary Figure S5). Only for some of the individual lipids, decided information is available about a potential role in the late development of the brain, in brain function in general or in infanthood and adolescence. Plasmalogens PC(O-42:0) and PC(O-38:3) serum levels were found indicative for predicting the development of celiac disease in infants at risk at the age of four months [66]. In general, plasmalogens that are a major component in the adult human brain, are only a minor component in the newborn with a steep increase in amount in the first postnatal year [67]. On the contrary, maternal undernutrition resulted in abnormal behavior in male rat offspring and increased amounts of ethanolamine-containing plasmalogens [68]. Therefore, it is difficult to evaluate the meaning of the increase in single plasmalogen species due to acitretin-treatment in the brain of young mice and to deduce possible implications for human infants and adolescents. A decrease in sphingomyelin C26:1, as observed here, was also reported in Elovl1 mutated infant/adolescent patients’ fibroblasts [69] and hypomyelination of central white matter explained the symptoms spastic paraplegia and central nystagmus. However, the total amount of sphingomyelin was non-significantly elevated upon acitretin injections, which might compensate for the lack of a single lipid species.

Lastly, the observation that TAG with saturated FAs were decreased upon acitretin-treatment might need to be addressed. Growing evidence suggests that mono- and polyunsaturated fatty acids are paramount for proper brain development and function (reviewed in [70]). For example, triglyceride-rich lipoproteins isolated from human blood after intake of a SFA-based diet led to microglia prone to an M1 phenotype, while those with monounsaturated fatty acids (MUFA) enhanced M2 differentiation, which could be confirmed for the brain of mice fed with respective diets [71]. Thus, the reduction in the saturated TAG species for about 25% might be classified as beneficial.

In sum, we here report that with subchronic, systemic acitretin administration, effects in the brain of adolescent/young adult mice remain limited and only single lipid species were affected, while liver tissue showed a more pronounced outcome. As we did not investigate spatial distribution of altered lipid profiles, we cannot exclude that single regions or cell types of the brain are specifically compromised by the synthetic retinoid in the late development of the brain. We also cannot deny that lipid metabolism of mice and man differs in some regard (e.g., [72,73,74]); nevertheless, observed effects do not point at a severe effect on late brain developmental stages and would advocate usage of acitretin in younger individuals. A close monitoring of lipid homeostasis referring to the more intense changes observed for liver remains advisable.

## 4. Materials and Methods

### 4.1. Treatment of Mice with Acitretin

Female C57Bl6/J OlaHsd mice (Envigo, Horst, Netherlands) were housed in groups of maximally five animals with free access to food and water (n = 6 animals per group, 10 weeks at the end of the treatment). A 12 h light–dark cycle (6 am to 6 pm light on) was maintained and a temperature of 22 °C and a relative humidity of 60% established. Experimenters were not blinded during drug injection but personnel dissecting brain was blinded towards the treatment. All experiments including animals were carried out in compliance with the ARRIVE guidelines (http://www.nc3rs.org.uk/page.asp?id=1357; accessed on April 2022) and all experimental procedures were carried out in accordance with the European Communities Council Directive regarding care and use of animals for experimental procedures and were approved by local authorities (LUA Rhineland-Palatinate; G14-1-087). Acitretin was prepared and administered as described before [32]. Control animals received the solvent (corn oil, Sigma-Aldrich, Darmstadt, Germany). Animals were sacrificed after isoflurane anesthesia and brains and liver dissected.

The left hemisphere and the right liver lobe were washed with distilled water and immediately stored at −20 °C (for long-term storage at −80 °C). For subsequent mass spectrometry experiments, homogenates of the complete left hemisphere or the right liver lobe were used.

### 4.2. RNA Extraction and qRT-PCR

RNA was extracted from one formalin-fixed liver lobe (Quick-RNA FFPE Kit, Zymo Research, Freiburg, Germany). Quantitative polymerase chain reaction (qPCR) was performed using exon–exon boundary-spanning primer sequences (see below) and the SYBR Green methodology on a Step One Plus sequence amplification system (Applied Biosystems, Foster City, CA, USA). The relative mRNA expression of the tested gene normalized to Gapdh expression was calculated using the ΔΔCt method. Primer sequences (primers obtained from Eurofins Genomics, Ebersberg, Germany) were as follows: Elovl3 forward/reverse GGACCTGATGCAACCCTATG/CCAACAACGATGAGCAACAG [75]; Lcat forward/reverse TATGTGATGGGGCTGCCT/GCTTGTGTTGTAGACAATCCTG [76]; D5d forward/reverse GGTGGCCTTGATGTGCTT/GCTATGCTTCCCGCTGAA.

### 4.3. Measurement of Different Lipid Species Using Mass Spectrometry

#### 4.3.1. Chemicals, Reagents, and Standards

High performance liquid chromatography (HPLC)-grade water, ethanol, and methanol were purchased from Fisher Scientific (Schwerte, Germany). HPLC-grade pyridine, phenyl isothiocyanate (PITC) and ammonium acetate were purchased from Merck (Darmstadt, Germany). The respective standards from Avanti Polar Lipids were used for normalization: 06:0 PC (DHPC), 19:0 Lyso PC, 08:0 PE, 06:0 SM (d18:1/6:0), and Splash^®^ II Lipidomix^®^ Mass Spec Internal Standard. The carnitine standards octanoyl-L-carnitine d3 and palmitoyl-L-carnitine d3 were purchased from Supelco Analytical (Munich, Germany). One standard for each lipid class was used for internal normalization allowing to over-come potential differences in the extraction efficiency during the lipid extraction (see Section 4.3.3). Moreover, these standards were essential for the calculation of potential matrix effects (see Section 4.3.4).

#### 4.3.2. Sample Preparation

The left hemisphere of mouse brain and the right liver lobes from six mice per group were mechanically homogenized in HPLC-grade water (Fisher Scientific, Schwerte, Germany) using Minilys (Peqlab, Erlangen, Germany) for 60 s on maximum intensity. Protein was measured using bicinchoninic acid assay according to [77] and homogenates were adjusted to a protein amount of 10 mg/mL in HPLC-grade water (Fisher Scientific, Schwerte, Germany).

#### 4.3.3. Lipid Extraction

The used solid/liquid lipid extraction method has been described in Grimm et al. (2011) [62]. Briefly, a 96 well filter plate (0.45 µm; Merck, Darmstadt, Germany) was fixed on a 96-deep well plate (Fisher Scientific) and circles of Whatman blotting paper (diameter of 6 mm) were placed into the wells of the filter plate. A standard mixture was added, followed by 10 µL of each prepared sample containing 100 µg protein (10 µg/µL). After drying the samples under a nitrogen flow, 20 µL of 5% PITC (*v*/*v*) diluted in ethanol/water/pyridine (1:1:1, *v*/*v*/*v*) were added to the wells and incubated for 20 min at room temperature. Samples were dried for 45 min under nitrogen and lipids were extracted by the use of 300 µL 4.93 mM ammonium acetate in HPLC-grade methanol for 30 min at 450 rpm on a plate shaker (IKA, Staufen, Germany). Liquid samples were transferred into the 96-deep well plate by centrifugation for 2 min at 500 g. Afterwards, samples were diluted with 600 µL 5 mM ammonium acetate in methanol/water (97:3, *v*/*v*; both HPLC-grade) before mass spectrometry analysis. An average extraction efficiency of > 80.7% (intra-day variance of 3.9%) and a linearity of R2 > 0.96 for this lipid extraction method were determined for these experimental conditions (Lauer et al., 2021 [32]).

#### 4.3.4. Targeted Shotgun Mass Spectrometry

For measurement of different species of diacyl-phosphatidylcholines (PC aa), phosphatidylcholine-plasmalogens (PC ae), lyso-phosphatidylcholines (Lyso PC), acyl- and acetyl-carnitines, sphingomyelins (SM) and triglycerides (TAG) a tandem mass spectrometry (also known as MS/MS) technique on a 4000-quadrupole linear-ion trap (QTrap) equipped with a Turbo Spray ion source (AB SCIEX, Darmstadt, Germany) was used as described previously [32]. A total of 20 µL per sample were injected via flow injection analysis with help of an auto-sampler of the Agilent HPLC 1200 series with a flow rate of 30 µL/min for 2.4 min, 200 µL/min from minute 2.4–2.8 and 30 µL/min from minute 2.8–3.0. One sample was analyzed two times (technical duplicates) using six biological replicates per group. Lipid analysis was performed in positive mode, with the individual parameters selected as follows: scan type = multiple reaction monitoring (MRM); measurement period = 3 min; curtain gas = 20.0 psi; collision gas = medium; ion spray voltage = 5500.0 V; temperature = 200.0 °C; ion source gas 1 = 40 psi; ion source gas 2 = 50 psi; inter-face heater = on; entrance potential = 10 V; collision cell exit potential = 15 V. The used Q1 and Q3 masses for MRM-mode as well as declustering potentials (DP) and collision energies (CE) for each metabolite were used as described in literature [32,78,79,80]. The specific conditions for each analyzed lipid species are listed in Supplementary Tables S1–S3, whereby Q1 masses range from 162.1–986.9 Da. The parameters were used in line with literature [32,78,79,80]. Parameters acquired from literature using a different MS were adjusted by ramping the parameters listed in Supplementary Table S3, in order to achieve a maximum in signal strength. The ramping was performed following the instructions of manual “Analyst Tutorial, Version 1.4” from Applied Biosystems and MDS Sciex. Detection of different lipid species was done using the Analyst 1.4.2 software (AB SCIEX, Darmstadt, Germany), whereby the average signal-to-noise ratio was 15.5 ± 2.1. An example of MS analysis/results is presented in Supplementary Figure S7. Potential matrix effects were evaluated by calculating the ratio between the deuterated lipid standards in presence of lipid extracts from acitretin-treated and control mice. The change in the ratio was maximum 3.94% and in average 1.31% (see Supplementary Figure S8). For an exemplary MS result see Supplementary Figure S9.

#### 4.3.5. Data Analysis and Statistical Analysis

Counts per second for each MRM pair were extracted via the Analyst 1.4.2 software (AB SCIEX, Darmstadt, Germany). Each lipid was normalized to its respective lipid class standard. After normalization, the mean per duplicate was formed for each lipid/standard ratio per mouse. Statistical analysis was carried out with R (R Core Team 2020; Vienna, Austria; https://www.R-project.org/, accessed on 1 June 2021). *p* value calculation for each parameter, shown in volcano plots, was carried out using two-tailed Student’s *t*-test. Volcano plots were created via the R package „EnhancedVolcano“(Kevin Blighe, Sharmila Rana and Myles Lewis (2020); version 1.6.0. https://github.com/kevinblighe/EnhancedVolcano, accessed on 1 June 2021). Statistical analysis of the average lipid class effect against the respective control was carried out via a two-tailed one-sample *t*-test. Error bar graphs represent standard error of the mean. Significance was set at * *p* ≤ 0.05, ** *p* ≤ 0.01 and *** *p* ≤ 0.001.

## Data Availability

All data are provided within the text or within Supplementary Material.

## Supplementary Materials

The following supporting information can be downloaded at: https://www.mdpi.com/article/10.3390/ijms232415535/s1.
