# Supplementary material for: The Influence of Acitretin on Brain Lipidomics in Adolescent Mice—Implications for Pediatric and Adolescent Dermatological Therapy
Source: Int J Mol Sci. 2022 Dec 8;23(24):15535. doi: 10.3390/ijms232415535 (PMC9778912; doi:10.3390/ijms232415535)
Supplement: Supplementary file 1 [file ijms-23-15535-s001.zip › Manuscript_IJMS_KE_09.22_Suppl. Figures_proofs.pdf]

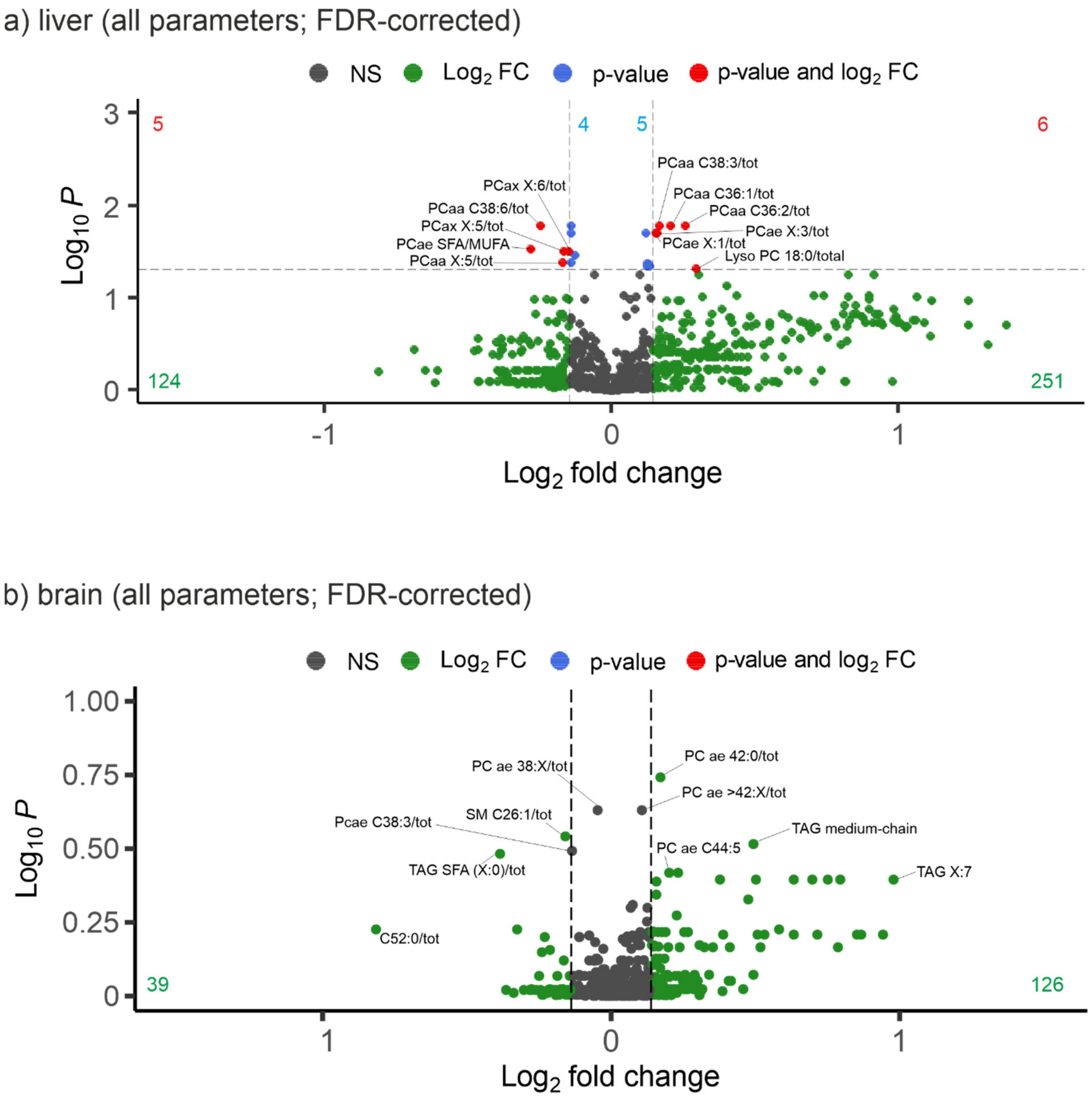

Suppl. Figure S1: Lipid changes in acitretin-treated wild type mice compared to controls after false discovery rate (FDR) correction. Mice were treated starting from the age of 9 weeks with seven daily acitretin i.p. injections. One liver lobe or one hemisphere of the brain were harvested for lipid extraction and analysis. Fold changes of the 703 included parameters are plotted logarithmically against the p value (-Log10) for liver (a) and brain (b) of female C57Bl6/J OlaHsd mice (n=6 per group). A detailed description of the structure of the volcano plots can be found in the legend of Figure 1.

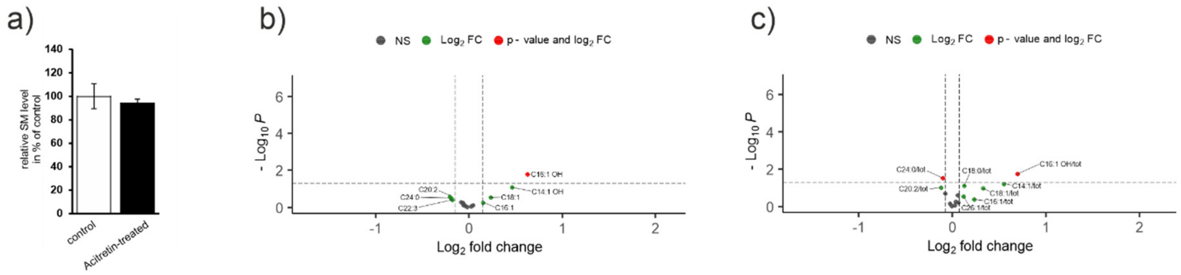

Suppl. Figure S2: SM in hepatic tissue of adolescent wild type mice after acitretin-treatment. a) Total amount of SM species in control and acitretin-treated hepatic tissue. Fold change and p-values for individual SM species is depicted in b). Alterations in lipid species that were significant after normalization to the lipid class are shown in c). Statistical significance of the mean effects in liver tissue of acitretin-treated mice compared to control mice was calculated using two-sample t-test (n=6 animals per group).

	liver	brain
PC ae C30:0	188.4 ± 15.3 % (*)	100.0 ± 9.6 %
PC ae C30:1	200.7 ± 19.7 % (*)	106.2 ± 11.7 %
PC ae C30:2	199.9 ± 19.6 % (*)	102.5 ± 17.3 %
PC ae C32:1	184.8 ± 15.9 % (*)	104.8 ± 2.2 %
PC ae C34:3	190.2 ± 17.1 % (*)	99.3 ± 10.1 %
PC ae C40:2	119.4 ± 4.8 % (*)	100.5 ± 4.7 %
PC ae C40:3	118.9 ± 4.6 % (*)	98.8 ± 5.2 %
PC ae C42:1	159.4 ± 14.9 % (*)	113.1 ± 2.9 %
PC ae C42:2	134 ± 7.6 % (*)	105.3 ± 2.9 %
Lyso PC C17:0	172 ± 13.4 % (*)	101.7 ± 6.4 %
Lyso PC C26:0	176.8 ± 15.6 % (*)	111.5 ± 9.1 %
Lyso PC C28:0	203 ± 16.3 % (*)	109.0 ± 9.9 %
Lyso PC C28:1	204.8 ± 19.5 % (*)	132.6 ± 15.4 %
SM C16:1 OH	154.8 ± 11.2 % (*)	97.8 ± 10.9 %
C03 OH	132.4 ± 4.4 % (*)	100.9 ± 9.3 %
C10	113.3 ± 4.4 % (*)	103.8 ± 4 %
PC aa C24:0/tot	207.7 ± 20.3 % (*)	90.6 ± 18.6 %
PC aa C28:0/tot	237.2 ± 18.3 % (*)	100.5 ± 14.2 %
PC aa C28:1/tot	208.6 ± 20.4 % (*)	95.6 ± 15.7 %
PC aa C34:3/tot	90.8 ± 3.1 % (*)	92.0 ± 3.2 %
PC aa C36:1/tot	115.4 ± 3.1 % (**)	100.6 ± 2.1 %
PC aa C36:2/tot	119.6 ± 2.8 % (**)	98.4 ± 1.9 %
PC aa C36:4/tot	90.7 ± 1.3 % (***)	96.3 ± 3.4 %
PC aa C38:3/tot	112.5 ± 1.7 % (*)	98.7 ± 1.8 %
PC aa C38:5/tot	86.9 ± 2.7 % (*)	96.1 ± 1.6 %
PC aa C38:6 /tot	84.3 ± 3 % (**)	98.9 ± 0.9 %
PC aa C40:5 /tot	90.7 ± 2.1 % (*)	105.4 ± 1.5 %
PC aaX:4/X:6	109 ± 2 % (**)	97.4 ± 2 %
PC aaX:4/(X5+X6)	109.7 ± 1.9 % (**)	98.4 ± 2 %
PC aa X:2/tot	106.3 ± 1.2 % (*)	99.3 ± 1.4 %
PC aa X:5/tot	88.8 ± 2.3 % (**)	98.3 ± 1.1 %
PC aa X:6/tot	90.8 ± 2.4 % (*)	102 ± 1.2 %
PC aa short (<32:X)/tot	183.3 ± 17.5 % (*)	98.2 ± 8.7 %
PC aa <30:X/tot	199.2 ± 18.7 % (*)	98.9 ± 16.1 %
PC aa 36:X/tot	103.8 ± 1.1 % (*)	99.3 ± 1.7 %
PC ae C30:0/tot	180.9 ± 14.3 % (*)	98.1 ± 10.5 %
PC ae C30:1/tot	191.3 ± 18.1 % (*)	106.1 ± 12.5 %
PC ae C30:2/tot	189.6 ± 18.1 % (*)	104 ± 18.2 %
PC ae C32:1/tot	175.4 ± 14.2 % (*)	100.8 ± 1.2 %
PC ae C32:2/tot	183.5 ± 18.4 % (*)	90 ± 7.4 %
PC ae C34:3/tot	179.3 ± 15.1 % (*)	99.5 ± 10.9 %
PC ae C38:0/tot	83.2 ± 3.3 % (**)	92.6 ± 2.2 %
PC ae C40:0/tot	88.9 ± 4.1 % (*)	98.7 ± 1.4 %
PC ae C42:0 /tot	120.2 ± 5.5 % (*)	112.6 ± 2.6 % (**)
PC ae C42:1 /tot	151.2 ± 12.4 % (*)	109.5 ± 3 %
PC ae C42:2 /tot	127.6 ± 6.1 % (*)	102.1 ± 4.1 %
PC ae MUFA	117.6 ± 5.1 % (*)	105.3 ± 2.8 %
PC ae SFA/MUFA	82.2 ± 5.1 % (**)	98.4 ± 1.2 %
PC ae SFA/PUFA	89.7 ± 3.6 % (*)	99.7 ± 1.5 %
PC ae MUFA /PUFA	109.9 ± 2.9 % (*)	101.2 ± 1 %

	liver	brain
PC ae X:2	114.4 ± 3.4 % (*)	103.6 ± 2.3 %
PC ae X:3	118.2 ± 5.2 % (*)	102.2 ± 2.9 %
PC ae short-chain < C32:X	198 ± 18.7 % (*)	102.4 ± 11.8 %
PC ae C32:X	186.8 ± 17 % (*)	103.7 ± 2.4 %
PC ae 42:X	130.6 ± 8.4 % (*)	109.1 ± 2.6 %
PC ae SFA / PC ae total	90.8 ± 2.8 % (**)	99.4 ± 0.8 %
PC ae MUFA / PC ae total	111.5 ± 2.7 % (**)	101 ± 0.6 %
PC ae X:2/tot	108.7 ± 1.9 % (**)	99.6 ± 0.9 %
PC ae X:3/tot	111.8 ± 2.9 % (**)	99 ± 2.4 %
PC ae 30:X/tot (PC ae short (<32:X)/tot)	188.8 ± 17.2 % (*)	101.8 ± 12.7 %
PC ae medium (32-36:X)/tot	107.2 ± 2.5 % (*)	99.7 ± 0.8 %
PC ae long (>36:X)/tot	96 ± 1.5 % (*)	100.2 ± 0.6 %
PC ae 32:X/tot	177.6 ± 15.3 % (*)	99.8 ± 1.4 %
PC ae 38:X/tot	91.7 ± 1.7 % (**)	96.8 ± 1.2 % (*)
PC ae 42:X/tot	123.6 ± 6.1 % (*)	105.3 ± 1.8 %
PC ae SFA/MUFA /aa	76.9 ± 9.8 % (*)	103 ± 2.6 %
PC ae C30:X / PC aa C30:X	174.2 ± 16.9 % (*)	96.1 ± 22.9 %
PC ae C32:X / PC aa C32:X	248.9 ± 19.3 % (*)	102 ± 13.7 %
PC axX:4/X:6	109.1 ± 2 % (**)	97.3 ± 1.4 %
PC axX:4/(X5+X6)	109.5 ± 1.8 % (**)	97.5 ± 1.4 %
PC ax X:2/tot	105.9 ± 1.1 % (*)	99.2 ± 1.6 %
PC ax X:5/tot	89.1 ± 2.1 % (**)	100.4 ± 1.6 %
PC ax X:6/tot	90.2 ± 2.5 % (**)	101.2 ± 2 %
PC ax short (<32:X)/tot	186.5 ± 17.5 % (*)	99.3 ± 10.1 %
PC ax 30:X/tot	163.2 ± 14.8 % (*)	97.3 ± 9.5 %
PC ax 36:X/tot	103.2 ± 1.1 % (*)	99.1 ± 1.3 %
PC ax 42:X/tot	135.4 ± 10.2 % (*)	104 ± 7.1 %
PC ax >42:X/tot	167.3 ± 15.1 % (*)	105.4 ± 6.2 %
PC ae/aa C30:0	192.9 ± 16.1 % (*)	94.9 ± 21.1 %
PC ae/aa C32:1	237.1 ± 18.5 % (*)	106.7 ± 12.8 %
PC ae/aa C32:2	216.2 ± 20.1 % (*)	92.1 ± 19.2 %
PC ae/aa C34:3	259.7 ± 20.6 % (*)	103.6 ± 19.9 %
Lyso PC C26:X	164.5 ± 15.6 % (*)	111.9 ± 10.5 %
Lyso PC C28:X	204 ± 17.7 % (*)	120.3 ± 12.4 %
Lyso PC 20:4/total (lyso PC X:4/total)	90.1 ± 2.5 % (*)	92.5 ± 6.4 %
Lyso PC 20:5/total (lyso PC X:5/total)	124.5 ± 3.7 % (*)	102.3 ± 7.5 %
Lyso PC 22:6/total (lyso PC X:6/total)	85.7 ± 4.3 % (*)	96.4 ± 6 %
Lyso PC 17:0/total (lyso PC 17:X/total)	177.6 ± 12.4 % (*)	91.8 ± 7.8 %
Lyso PC 24:0/total (lyso 24:X/total)	175.8 ± 16.9 % (*)	111.9 ± 15.2 %
Lyso 26:X/total	171.8 ± 16 % (*)	101 ± 14.5 %
Lyso 28:X/total	213.4 ± 18.2 % (*)	106.3 ± 14.8 %
Lyso PC 18:0/total	122.7 ± 3.5 % (**)	100.2 ± 2.1 %
Lyso PC 26:0/total	186.3 ± 15.8 % (*)	101.2 ± 12.5 %
Lyso PC 28:0/total	209.6 ± 16.5 % (*)	96.4 ± 11.7 %
Lyso PC 28:1/total	216.6 ± 20.2 % (*)	117.1 ± 18.1 %
SM C16:1 OH/tot	162.4 ± 12.7 % (*)	91.5 ± 5.7 %
SM C24:0/tot	93.4 ± 1.5 % (*)	99.2 ± 3.1 %
SM MUFA/tot	104.8 ± 1.5 % (*)	99.5 ± 2.6 %
C3:X	121.7 ± 4.2 % (*)	115.6 ± 10.4 %
C0/(C2+C3)	82.9 ± 5.5 % (*)	103 ± 6 %

Suppl. Figure S3: Significant changes of single lipid species in liver tissue upon acitretin-treatment. Elevated species are indicated in green, decreased species are labelled in red. Significance: *, $p<0.05$; **, $p<0.001$ as determined by Student's t-test. For a direct comparison, corresponding values obtained for brain tissue are given.

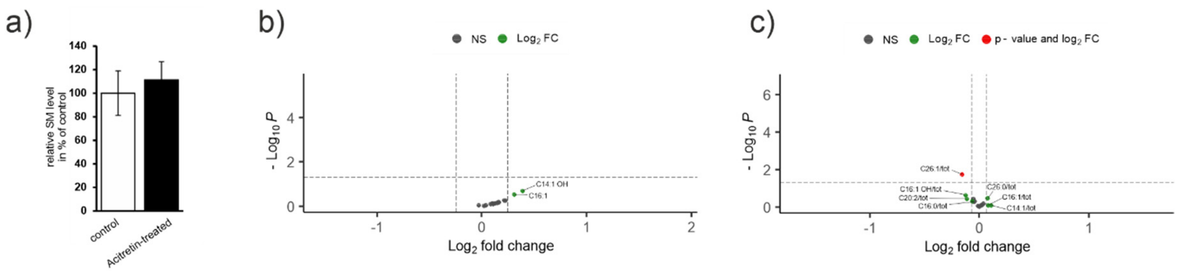

Suppl. Figure S4: SM in brain tissue of adolescent wild type mice after acitretin-treatment. a) Total amount of SM species in control and acitretin-treated hepatic tissue. Fold change and p-values for individual SM species is depicted in b). Changes in lipid species that were significant after normalization are shown in c. Statistical significance of the mean effects in liver tissue of acitretin-treated mice compared to control mice was calculated using two-sample t-test (n=6 animals per group).

	brain	liver
PC ae C42:0	117.5 ± 4.6 % (*)	127.0 ± 7.5 %
PC ae C44:5	115.0 ± 3.8 % (*)	121.3 ± 9.0 %
PC ae C38:3/tot	91.0 ± 2.4 % (*)	101.9 ± 1.9 %
PC ae C42:0/tot	112.6 ± 2.6 % (**)	120.2 ± 5.5 % (*)
PC ae C44:5 / PC aa 44:12/tot	110.7 ± 3.7 % (*)	114.1 ± 6.4 %
PC ae > 42:X	111.5 ± 2.3 % (*)	139.9 ± 12.1 %
PC ae 38:X/tot	96.8 ± 1.2 % (*)	91.7 ± 1.7 % (**)
PC ae >42:X/tot	107.7 ± 2.4 % (*)	132.2 ± 9.9 %
PC ax >42:X	111.5 ± 2.3 % (*)	139.1 ± 12.7 %
PC ax C42:0	117.1 ± 4.5 % (*)	118.7 ± 8.0 %
SM C26:1/tot	89.6 ± 3.2 % (*)	108.8 ± 6.0 %
TAG SFA (X:0)/tot	76.6 ± 7.0 % (*)	71.9 ± 7.5 %

Suppl. Figure S5: Significant changes of single lipid species in brain tissue upon acitretin-treatment. Elevated species are indicated in green, decreased species are labelled in red. Significance: *, $p<0.05$; **, $p<0.001$ as determined by Student's t-test. For a direct comparison, corresponding values obtained for liver tissue are given.

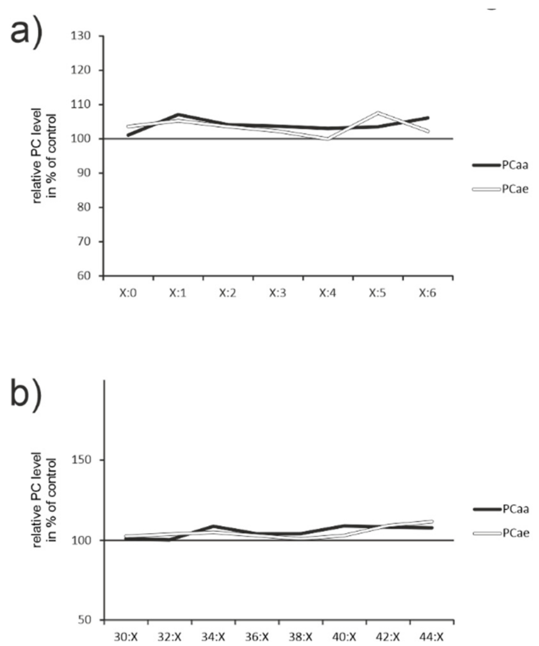

Suppl. Figure S6: Saturation and chain length of phosphatidylcholines in brain tissue from acitretin-treated mice. Processing of the data regarding saturation (a) and chain length (b) included the following spe-cies: PCaa saturation: C20-48:0, C28-42:1, C30-42:2, C32-40:3, C34-42:4, C36-42:5, C36-42:6; PCaa chain length: C30:0-2; C32:0-3; C34:1-4; C36:0-6; C38:0-6; C40:0-6, C42:0-6; C44:0; PCae saturation: C30-42:0; C30-42:1, C30-42:2; C34-44:3; C36-44:4; C36-44:5; C38-44:6; PCae chain length: C30:0-2; C32:1-2; C34:0-3; C36:0-5; C38:0-6; C40:0-6; C42:0-5; C44:3-6.

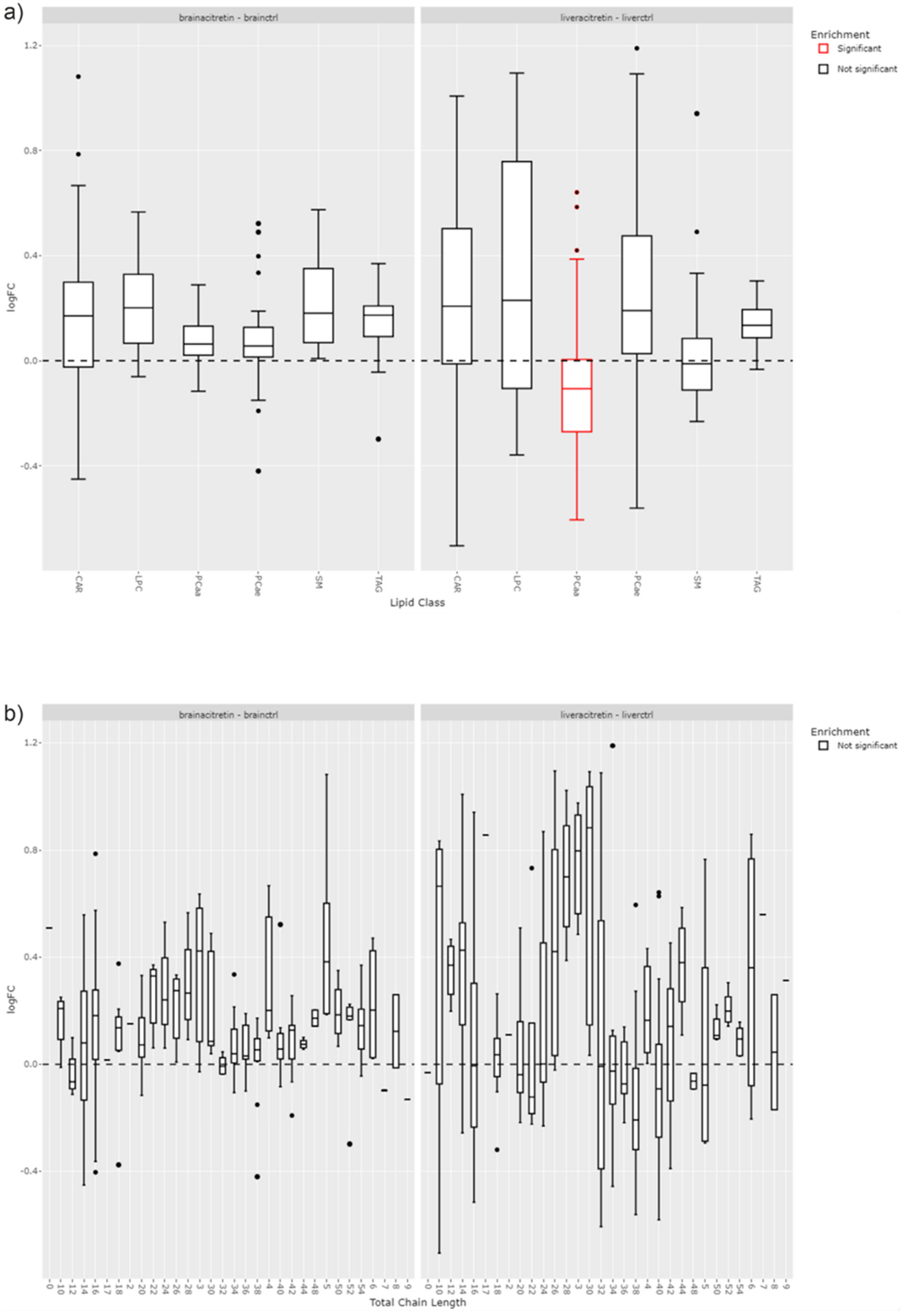
a)
brainacitretin - brainctrl
liveracitretin - liverctrl
Enrichment
Significant
Not significant
logFC
1.2
0.8
0.4
0.0
-0.4
CAR
LPC
PCaa
PCae
SM
TAG
Lipid Class
b)
brainacitretin - brainctrl
liveracitretin - liverctrl
Enrichment
Not significant
logFC
Total Chain Length

c)

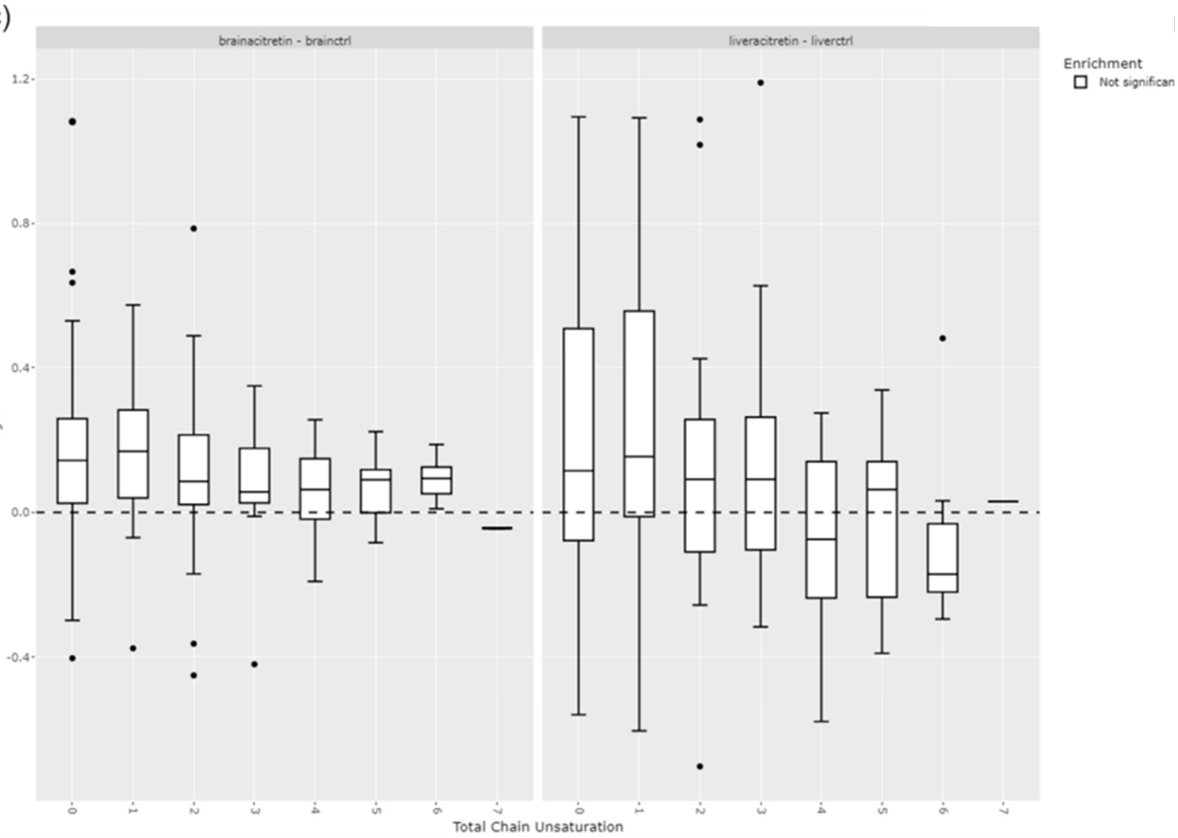
brainacitretin - brainctrl
liveracitretin - liverctrl
Enrichment
Not significan
logFC
Total Chain Unsaturation

d)

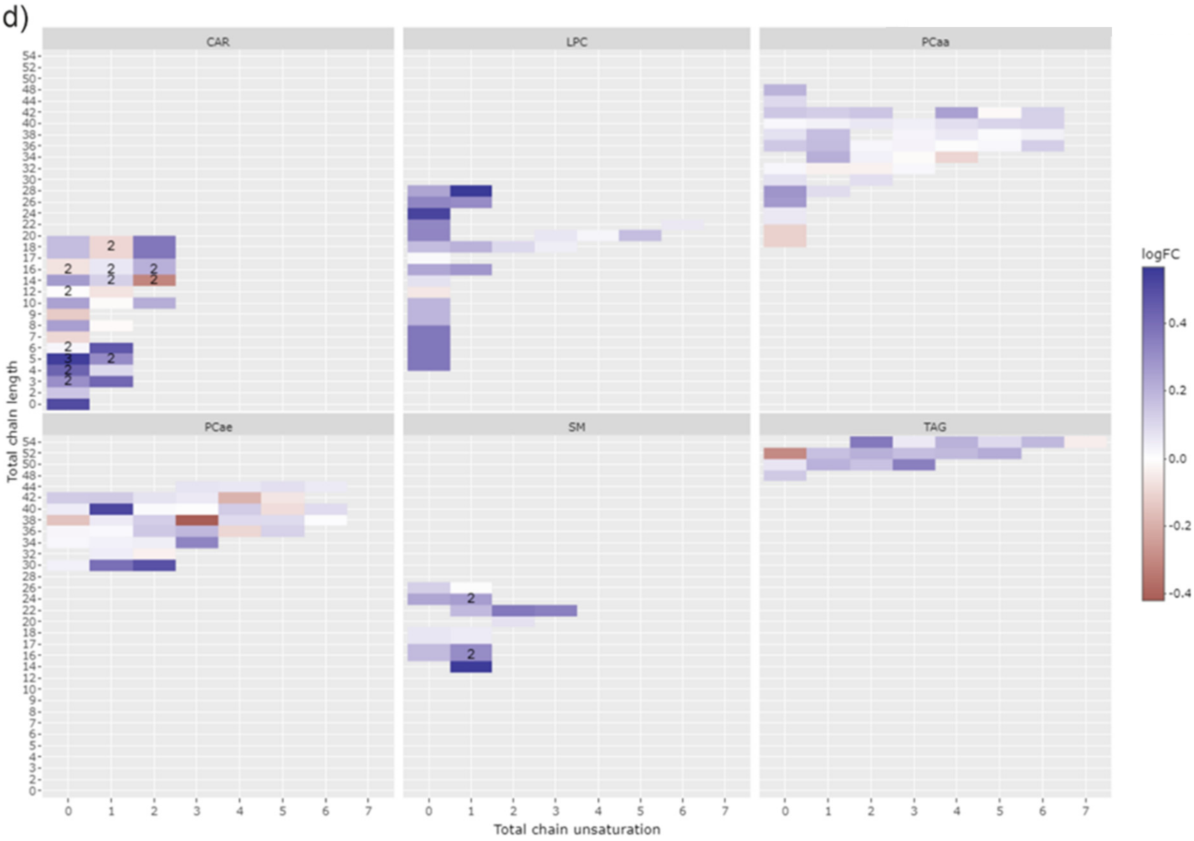
CAR
LPC
PCaa
PCae
SM
TAG
logFC
Total chain length
Total chain unsaturation

e)

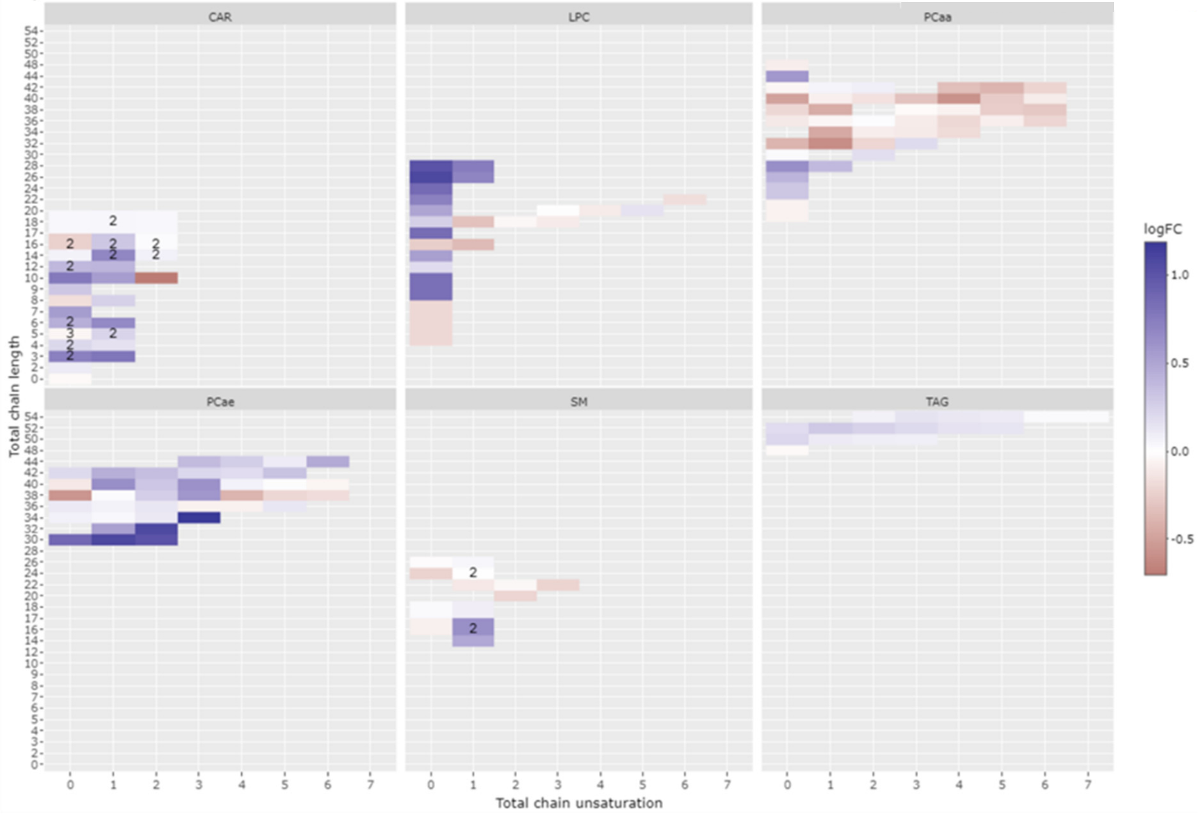

Suppl. Figure S7: Enrichment analysis using the lsea function from the R package "lipidr". (Mohamed A, Molendijk J (2020). _lipidr: Data Mining and Analysis of Lipidomics Datasets_. R package version 2.10.0, <https://github.com/ahmohamed/lipidr>.) (a) Visualization of enrichment analysis (EA) result categorized by lipid class. Left side showing effect of acitretin on brain against control, right side indicating liver tissue. Boxplot in red indicating significant difference between acitretin and control. (X-axis sorted by lipid class, Y-axis logFC of acitretin against corresponding control). (b) EA sorted by chain length. (c) EA sorted by saturation. (d) Fold change of lipids per class showing total chain length and unsaturation of the acitretin-effect on brain tissue against control. Numbers on the plot indicate multiple lipids measured with the same chain properties. (e) Fold change of lipids per class showing total chain length and unsaturation of the acitretin-effect on liver tissue against control. Numbers on the plot indicate multiple lipids measured with the same chain properties.

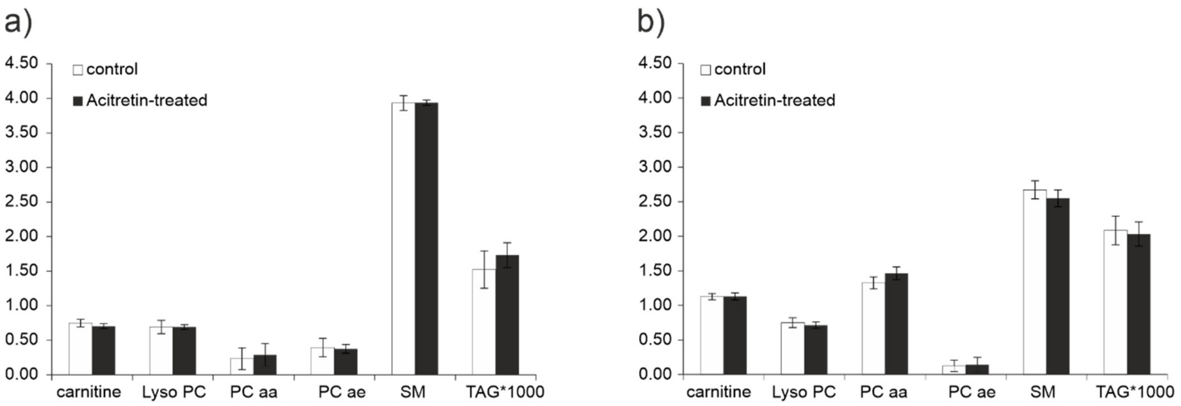

Suppl. Figure S8: Calculated matrix effects of the measured parameters carnitine, Lyso-Phosphatidylcholine (Lyso PC), Phosphatidylcholine (PCaa), Phosphatidylcholine-Plasmalogens (PCae), Sphingomyelines (SM) and Triacylglycerides (TAG) in control and acitretin-treated mice in a) liver and b) brain tissue.

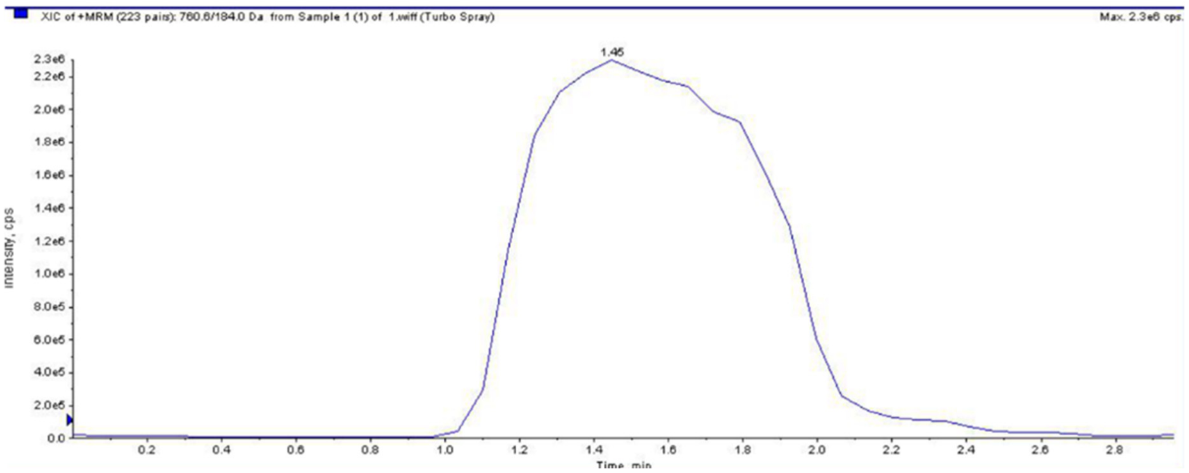

Suppl. Figure S9: An example of MS analysis/ result.
